# The effect of health and nutrition education intervention on women's postpartum beliefs and practices: a randomized controlled trial

**DOI:** 10.1186/1471-2458-9-45

**Published:** 2009-02-01

**Authors:** Nian Liu, Limei Mao, Xiufa Sun, Liegang Liu, Ping Yao, Banghua Chen

**Affiliations:** 1 Department of Nutrition and Food Hygiene, School of Public Health, Tongji Medical College, Huazhong University of Science & Technology, 13 Hangkong Road, Wuhan 430030, PR China; 2 Hubei Maternal and Child Health Hospital, 745 Wuluo Road, Wuhan 430070, PR China; 3 Department of Nutrition and Food Hygiene, School of Public Health and Tropical Medicine, Southern Medical University, Guangzhou 510515, PR China

## Abstract

**Background:**

'Sitting month' is the Chinese tradition for postpartum customs. Available studies indicate that some of the traditional postpartum practices are potentially harmful for women's health. However, no intervention study aiming at postpartum practices has been performed. In this paper we evaluated the effect of a health and nutrition education intervention, which focused on improving postpartum dietary quality and optimal health behaviors.

**Methods:**

The study design was a randomized controlled trial conducted in both urban and rural area of Hubei between August 2003 and June 2004. A total of 302 women who attended the antenatal clinic during the third trimester with an uncomplicated pregnancy were recruited. Women randomized to the education intervention group in both urban and rural area received two two-hour prenatal education sessions and four postpartum counseling visits. Control group women received usual health care during pregnancy and postpartum period. Women were followed up until 42 days postpartum. Outcome measures were nutrition and health knowledge, dietary behavior, health behavior and health problems during the postpartum period.

**Results:**

Women in the intervention groups exhibited significantly greater improvement in overall dietary behaviors such as consumption of fruits, vegetables, soybean and soybean products as well as nutrition and health knowledge than those in the control groups. Significantly more women in the intervention groups give up the traditional behavior taboos. The incidence of constipation, leg cramp or joint pain and prolonged lochia rubra was significantly lower in the intervention groups as compared with the control groups.

**Conclusion:**

The study shows that health and nutrition education intervention enable the women take away some of the unhealthy traditional postpartum practices and decrease the prevalence of postpartum health problems. The intervention has potential for adaptation and development to large-scale implementation.

**Trial registration number:**

klACTRN12607000549426

## Background

Although much educational intervention has focused on pregnancy related nutrition and health problems [1-3]. Such education programs are often not maintained during the postpartum period. Postpartum period received less attention as compared with pregnancy and childbirth [4].

The postpartum period, or puerperium, starts about an hour after the delivery of the placenta and includes the following six weeks [5]. By six weeks after delivery, most of the changes of pregnancy, labor, and delivery have resolved and the body has reverted to the nonpregnant state [5,6]. The postpartum period is a very special phase in the life of a woman. Her body needs to heal and recover from pregnancy and childbirth. A good postpartum care and well balanced diet during the puerperal period is very important for the health of a woman.

According to Chinese traditions, the first 30 or 40 days postpartum is recognized as a special time period for behaviour restrictions and a state for convalescence. This period is called 'sitting month' or 'doing the month'. Based on Chinese traditional medicine, postpartum women are in a 'weak' state because of 'Qi' deficiency and blood loss [7]. Their body can be easily attacked by 'heat' or 'cold ', which may cause some health problems like dizziness, headache, backache and arthragia in the month or in later years. Therefore, Chinese women are advised to follow a specific set of food choices and health care practices. For example, the puerperal women should stay inside and not go outdoors; all windows in the room should be sealed well to avoid wind. Bathing and hair washing should be restricted to prevent possible headache and body pain in later years. Foods such as fruits, vegetables, soybean products and cold drinks which are considered 'cold', should be avoided [8,9]. In contrast, foods such as brown sugar, fish, chicken and pig's trotter which are considered 'hot' should be encouraged [9,10]. It is believed that if a woman does not observe these restrictions, she may suffer a poor health at her later life.

Several studies indicated that the incidences of postpartum health problems are high and these problems maybe have relation to traditional and unscientific dietary and behavior practices in the postpartum period [11-13]. Available Chinese data also suggested that the incidences of constipation and hemorrhoids were associated with lack of exercises and a decreased intake of fruit and vegetables, the risk of oral problems were associated with not brush the teeth and excessive intake of sugar during the puerperium [11,13].

This article describes a health and nutrition education intervention undertaken in both urban and rural area to try to overcome the traditional unhealthy postpartum practices. This paper builds on an earlier report where it was shown that the traditional postpartum convalescence habits mainly passed down from senior females in the family to the younger generations. Women have limited access to contemporary postpartum practices, especially those who living in rural area [14]. The goals of the intervention were to provide information and guidance on contemporary postpartum practices and take away common misconceptions about traditional dietary and health behaviors (e.g. fruit and vegetables should be restricted because of cold nature, postpartum women should stay inside and not go outdoors). In order to allow a decision to be made about possible larger scale implementation of this intervention, the present study aimed at assess the effects of the intervention on dietary and health behavior among the participants.

## Methods

### Study sites and participants

People's life styles varied considerably between metropolitan and rural areas. The rural areas remain underdeveloped economically and reserved culturally. Thus two regions representing urban and rural areas in Hubei province have been selected respectively. One is in urban area of Wuhan, which is the provincial capital of Hubei with a population of 7 000 000. The other is in rural area of Macheng, a county located 200 km east of Wuhan with a population of 1 200 000. The study was carried out between August 2003 and June 2004. The participants were selected from four hospitals in the urban area and four health centers in the rural area, respectively. The eligibility criteria for enrollment were as follows: 1) healthy pregnant women; 2) at their third trimester; 3) had at least three routine examinations at these antenatal clinics. A total of 410 women were originally enrolled in the study. Information on study procedures was provided to eligible pregnant women. Each woman signed a consent form at the first time interview, confirming her willingness to participate. Ethics approval was obtained from the local Health Department and the research ethics boards of Tongji Medical College, China.

### Intervention study design

This study was designed as a randomized controlled trial to evaluate the effectiveness of a health and nutrition education intervention with the main focus on dietary quality and health behavior during the puerperium. Participating women were randomly assigned to either intervention or control group in both urban and rural areas. Women of the two intervention groups in both areas were informed to take part in two, two-hour health and nutritional education sessions. The sessions were held twice a month to ensure that every participant in the intervention groups could join in. The health educators were health staffs from the local maternal and child hospital, who were intensively trained by our research team. The content of the two sessions focused on:

• Food guide pyramid knowledge

• Nutrient-food association knowledge

• The importance of dairy consumption, fruit and vegetable consumption

• Examples of healthy menus

• Optimal hygiene behavior and physical exercise patterns during postpartum period

• Discussions about healthy lifestyle during postpartum period

• Common misconceptions on hygiene behaviours during puerperium

• Discussions about nutrition and health problems concerns postpartum practices

**Figure 1:**
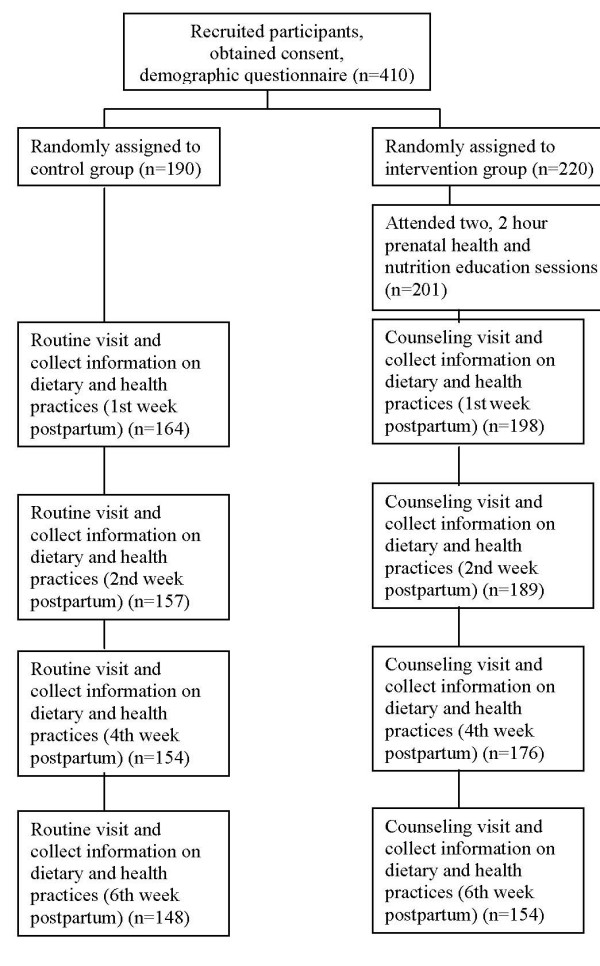
**Schematic diagram of study design**.

A guidebook concerning postpartum nutrition and health care knowledge which was compiled by our research team was disseminated to the intervention group women after class. Participants in the control groups didn't receive the intervention but were exposed to the normal standard of care available during the delivery and postpartum period. Figure 1 shows the trial profile.

### Data collection

A baseline questionnaire including socio-demographic characteristics of the households were collected prenatally at the time of the recruitment. The nutrition and health knowledge test was performed for all the participants at the same time. During the puerperium, the trained health staffs made four visits to the households of each participant at 1, 2, 4 and 6 weeks postpartum. During each home visit, a questionnaire was used to interview the women in the intervention and control groups in both areas. The questionnaire included information on their postpartum health behaviors, physical activities and health problems. The women and her infants were examined by the health professional. For the intervention group women in both areas, counseling and advice on postpartum practices were provided. For the participants in two control groups, the health professionals did not interfere with their practices, only routine examinations were provided. All the participants were asked to record consecutive individual 3-day food records of the first week, 2-day the following week, 2-day the fourth week and 2-day the sixth week postpartum. They were instructed on how to estimate food quantities in grams and milliliters at the initial visit. Dietary intakes were assessed by the 9 days food records. The same health staff made the all four-time repeated visits for each participant to minimize between-interviewer variation. The questionnaires including the nutrition and health knowledge test were designed by the scientists of our research team [see Additional file 1]. They were based on a previous research on women's postpartum practices [14]. Before the formal investigation, the questionnaires were pretested and revised repeatedly according to the analysis of the results.

### Analyses

Chinese food ingredients table was used to calculate the energy and nutrients of the food intakes [15]. Intake of nutrients was estimated and compared with the Chinese Recommended Nutrient Intake (RNI) [16]. Mean nutrient intakes for the diet records were calculated by averaging the intakes from the 9 days records. All statistical analyses were performed using SAS 8.1 statistical software package. Frequencies were used to describe characteristics of participants with respect to demographics. Participants in the intervention and control groups were compared using Chi-square and *t *tests.

## Results

### Characteristics of the participants

Figure 1 shows the participant flow diagram. Of the 410 women that agreed to participate in the study, 19 didn't participate all the lectures, 36 didn't finish all the dietary records, 53 dropped out during the study. The final analysis was completed with 302 women, with 154 in the intervention and 148 in the control groups. The age range of the samples was from 20 to 38 years, with a mean age of 26.33 ± 3.60 years in the urban and 26.28 ± 3.21 years in the rural area. The majority of the participants in the urban area had completed middle or high school education. The rural participants mainly received primary or middle school education and were predominately farmers. Table 1 shows the characteristics of the study participants. The differences between the intervention and control groups in both areas in terms of age, education and occupation were not substantial and not statistically significant.

**Table 1:** Characteristics of the participants

	Urban n = 142		Rural n = 160		Total n = 302	
	
	Intervention	Control	Intervention	Control	Intervention	Control
	n = 73 (%)	n = 69 (%)	n = 81 (%)	n = 79 (%)	n = 154 (%)	n = 148 (%)
**Age (years)**						
< 25	21(28.77)	19(27.54)	26(32.10)	28(35.44)	47(30.52)	47(31.76)
25–30	45(61.64)	38(55.07)	43(53.09)	43(54.43)	88(57.14)	81(54.73)
> 30	7(9.59)	12(17.39)	12(14.81)	8(10.13)	19(12.34)	20(13.51)
**Education**						
Primary school	4(5.84)	2(2.90)	18(22.22)	21(26.58)	22(14.29)	23(15.54)
Middle school	21(28.77)	24(34.78)	52(64.20)	46(58.23)	73(47.40)	70(47.30)
High school	18(24.66)	27(39.13	9(11.11)	11(13.92)	27(17.53)	38(25.68)
College	30(41.10)	16(23.19)	2(2.47)	1(1.27)	32(20.78)	17(11.48)
**Occupation**						
Laborer	12(16.44)	18(26.09)	14(17.28)	1(1.27)	26(16.88)	19(12.84)
Farmer	2(2.74)	1(1.45)	59(72.84)	73(92.41)	61(39.61)	74(50.0)
Technical	8(10.96)	3(4.35)	2(2.47)	1(1.27)	10(6.49)	4(2.70)
Government official	11(15.07)	5(7.25)	0	1(1.27)	11(7.14)	6(4.05)
Trader	4(5.48)	2(2.90)	0	1(1.27)	4(2.60)	3(2.03)
House duty	36(49.32)	40(57.97)	6(7.41)	2(2.53)	42(27.27)	42(28.38)
**Delivery way**						
Vaginal delivery	35(47.95)	27(39.13)	27(33.33)	41(51.90)	62(40.26)	68(45.95)
Cesarean section	38(52.05)	42(60.87)	54(66.67)	38(48.10)	92(59.74)	80(54.05)
**Parity**						
Primiparous	67(91.78)	63(91.3)	66(81.48)	68(86.08)	133(86.36)	131(88.51)
Multiparous	6(8.22)	6(8.70)	15(18.52)	11(13.92)	21(13.64)	17(11.49)

### Knowledge about nutrition and health care

The nutrition and health knowledge test was performed for all the participants at the time of the recruitment. The understanding rate of the nutrition and health knowledge was very low among all the participants. No significant differences were found between the intervention and control group in both areas before the intervention. The repeated test was performed at the last postpartum visit for all of them. Women in the intervention groups exhibited significantly great improvement in overall nutrition and health knowledge after the education sessions. In both areas, significantly more women in intervention groups responded correctly to the questions than those in the control groups. After the education sessions, most of the intervention group women knew that they could eat fruit and vegetables, brush teeth or take a bath during the puerperium. The results are shown in Table 2.

**Table 2:** Number of women who correctly answered questions on nutrition and health knowledge (Test performed at the last postpartum visit)

	Urban				Rural				Total			
	
	Intervewntion		Control		Intervention		Control		Intervention		Control	
	(n73)		(n = 69)		(n = 81)		(n = 79)		(n = 154)		(n = 148)	
	
	n	%	n	%	n	%	n	%	n	%	n	%
Milk is the best source of calcium	65	89.04	25	36.23**	45	55.56	29	36.71*	110	71.43	54	36.49**
Chicken is more nutritious than chicken soup	63	86.3	26	37.68**	55	67.9	30	37.97**	118	76.62	56	37.84**
Women can eat vegetables and fruit during puerperium	69	94.52	46	66.67**	70	86.42	51	64.56**	139	90.26	97	65.54**
Which food contains abundant vitamin C and carotene	67	91.78	31	44.93**	72	88.89	51	64.56**	139	90.26	82	55.41**
Food sources of high quality protein	26	35.62	6	8.70**	12	14.81	11	13.92	38	24.68	17	11.49**
Which food is iron-rich	63	86.3	40	57.97**	59	72.84	39	49.37**	121	78.57	79	53.38**
Kelp contains affluent iodine	69	94.52	54	78.26*	76	93.83	57	72.15**	145	94.16	111	75.00**
Colostrums should feed the infant	71	97.26	49	71.01**	77	95.06	53	67.09**	148	96.1	102	68.92**
Breast milk is the best food for infants	69	94.52	61	88.41	73	90.12	63	79.75*	142	92.21	124	83.78**
Bedroom should be ventilated everyday	68	93.15	51	73.91**	75	92.59	59	74.68**	143	92.86	110	74.32**
Relevant activity is better for recovery	69	94.52	54	78.26**	77	95.06	64	81.01**	146	94.81	118	79.73**
Sexual activity should not be initiated until 6 weeks after giving birth	68	93.15	44	63.77**	72	88.89	48	60.76**	140	90.91	92	62.16**
Vitamin D can be gained by basking in the sunshine	60	82.19	28	40.58**	66	81.48	41	51.90**	126	81.82	69	46.62**
Breastfeeding will not induce obesity of mother	67	91.78	50	72.46*	73	90.12	54	68.35**	140	90.91	104	70.27**
Women can brush teeth and take shower during puerperium	69	94.52	47	68.12**	73	90.12	55	69.62**	142	92.21	102	68.92**

** Indicates P-value of < 0.01 from *x*^2^-test between intervention and control groups.

* Indicates P-value of < 0.05 from *x*^2^-test between intervention and control groups.

### Dietary behaviour

Table 3 shows the mean daily intake of food categories of the participants. A significant intervention effect on soybean and soybean product, vegetables and fruit intake was found in urban area. Although fruit consumption in the rural area was very low, participants in the intervention group consumed significant more fruit than those in the control group. No changes in other food intakes were detected.

**Table 3:** Mean daily intakes of food categories of the participants (g/d/person)

	Urban		Rural		Total	
	
	Intervention (n = 73)	Control n = 69(%)	Intervention (n = 81)	Control n = 79(%)	Intervention (n = 154)	Control n = 148(%)
Grain or cereal	313.42	319.79	380.91	383.25	347.17	351.52
Egg	99.76	110.54	211.77	231.10	155.77	170.82*
Dairy	66.48	54.04	4.25	2.31	35.37	29.15*
Meat, poultry and Fish	272.73	275.91	496.69	458.9	384.71	367.41*
Soybean and soybean product	38.55	18.42**	183.16	140.90	110.86	79.66**
Vegetable	145.59	112.97*	410.41	414.22	278.01	263.60*
Fruit	75.71	44.74*	31.12	15.43**	53.42	30.09**

**Indicates *P*-value of < 0.01 from *t *test between intervention and control groups.

* Indicates *P*-value of < 0.05 from *t *test between intervention and control groups.

Significantly higher mean Vitamin C, Vitamin A and calcium intake were found in the intervention group as compared to the control group in urban area, but the intake of vitamin C and calcium were still remarkably below the Recommended Nutrient Intake. No significant differences were found in energy and nutrient intake between intervention and control group in the rural area (see Table 4).

**Table 4:** Mean energy and nutrients intakes of the study women

	Urban		Rural		Total	
	
	Intervention	Control	Intervention	Control	Intervention	Control
	(n = 73) (RNI%)	(n = 69) (RNI%)	(n = 81) (RNI%)	(n = 79) (RNI%)	(n = 154) (RNI%)	(n = 148) (RNI%)
Energy (kcal)	2381.04(91.57)	2344.54(90.17)	3306.15(129.08)	3240.12(126.54)	2843.60(110.33)	2792.33(108.36)
Protein (g)	88.93(104.62)	86.05(101.23)	156.24(183.82)	148.91(175.18)	122.59(144.22)	117.48(138.21)*
Retinol equivalent (μg)	813.16(67.76)	752.65(62.72)	2030.67(169.22)	2079.98(173.33)	1421.92(118.49)	1416.32(118.03)
Vit B1 (mg)	1.24(69.03)	1.15(64.16)	2.29(127.45)	2.09(116.05)	1.77(98.24)	1.62(90.11)*
Vit B2 (mg)	0.99(58.12)	0.96(56.59)	1.73(101.52)	1.71(100.71)	1.36(79.82)	1.34(78.65)
Vit PP (mg)	20.81(115.63)	19.76(109.80)	35.17(195.41)	33.62(186.79)	27.99(155.52)	26.69(148.3)*
Vit C (mg)	54.61(42.01)	41.69(32.07)*	123.20(94.77)	129.91(99.93)	88.91(68.39)	85.80(66.0)
Vit E (mg)	16.05(114.67)	14.13(100.90)*	33.54(239.58)	30.80(220.01)	24.80(177.13)	22.47(160.46)*
Calcium (mg)	481.25(40.10)	417.99(34.83)*	978.22(81.52)	914.42(76.20)	729.74(60.68)	666.21(55.52)*
Iron (mg)	21.74(86.98)	20.65(82.59)	37.50(150.01)	36.31(145.23)	29.62(118.50)	28.48(113.91)
Zinc (mg)	12.45(57.90)	11.95(55.59)	21.28(98.96)	20.43(95.03)	16.87(78.43)	16.19(75.49)

* Indicates *P*-value of < 0.05 from *t *test between intervention and control groups.

### Health behaviour

Scoring method was used to reflect the hygiene behaviors and physical activity during the puerperium. The details of the behaviours were recorded during the 1, 2, 4 and 6 weeks postpartum visit. Take exercising as an example, three points were awarded for exercising daily, two points were awarded for exercising more than three times per week, one point was awarded for exercising one to three times per week, no point was awarded to no exercise. Hygiene behaviors included the following five items: exposing in sunshine, bathing, hair washing, cleaning the perineum and ventilating the room. The accumulated score of the items for the four weeks altogether was the final score. The highest score of hygiene behavior was 60. Activity behavior included three items: exercising, participating in outdoor activities and doing household duties. The highest score of activity behavior was 36.

There is no significant difference on both hygiene score and activity score between the urban intervention and urban control group (urban intervention: 31.75 ± 5.94 and 5.76 ± 3.88; urban control: 31.23 ± 6.22 and 4.77 ± 3.61, P > 0.05). But the score on exercising were significantly higher in the urban intervention than the urban control group (intervention: 2.16 ± 2.53, control 1.18 ± 2.14, P < 0.05). The two scores of the rural intervention group were significantly higher than the control group(rural intervention: 37.03 ± 5.93 and 12.94 ± 7.00; rural control: 29.53 ± 10.17 and 7.27 ± 5.29, P < 0.05).

### Health problems during the puerperium

Table 5 shows the types of health problems reported during the puerperium. The most common problem was breast problems (41.06%) followed by prolonged rubra lochia (24.83%) and insufficient milk production (18.21%). The incidence of constipation, leg cramp or joint pain and abdominal pain was significantly lower in the urban intervention group than the control group. Significantly more women in the rural control group had prolonged lochia rubra than the intervention group. No significant differences were found in other health problems between the two groups in both areas.

**Table 5:** Incidence of maternal health problems during the puerperium

	Urban				Rural				Total			
	
	Intervention		Control		Intervention		Control		Intervention		Control	
	(n = 73)		(n = 69)		(n = 81)		(n = 79)		(n = 154)		(n = 148)	
	
	n	%	n	%	n	%	n	%	n	%	n	%
Constipation	12	16.44	22	31.88*	7	8.64	9	11.39	19	12.34	31	20.95*
Hemorrhoids	7	9.59	8	11.59	2	2.47	2	2.53	9	5.84	10	6.76
Anal fissure	2	2.74	3	4.35	1	1.23	3	3.8	3	1.95	6	4.05*
Urinary system infection	2	2.74	6	8.7	3	3.7	2	2.53	5	3.25	8	5.41
Cold or fever	5	6.85	6	8.7	1	1.23	3	3.8	6	3.9	9	6.08
Backache	21	28.77	18	26.09	1	1.23	0	0	22	15.58	18	12.16
Leg cramp or joint pain	0	0	5	7.25*	1	1.23	4	5.06	1	0.65	9	6.08**
Abdominal pain	2	2.74	12	17.39*	2	2.47	4	5.06	4	2.6	16	10.81**
Dizziness	4	5.48	10	14.49	0	0	2	2.53	4	2.6	12	8.11**
Headache	5	6.85	4	5.8	0	0	1	1.27	5	3.25	5	3.38
Oral inflammation	4	5.489	2	2.9	24	29.63	19	24.45	28	18.18	21	14.19
Gingival bleeding	6	8.22	3	4.35	10	12.13	11	13.92	16	10.39	14	9.46
Breast problems	43	58.9	38	55.07	3	3.7	12	15.19	46	29.87	50	33.78
Insufficient milk production	14	21.54	20	25.97	7	8.64	9	11.39	21	13.64	29	19.59*
Prolonged lochia rubra	29	39.73	31	44.93	2	2.47	2	2.53*	31	20.13	33	22.3

* Indicates *P*-value of < 0.05 from *t *test between intervention and control groups.

• Breast problems include breast swelling or pain, nipple fissure and mastitis.

• Insufficient milk production means the mother's milk secretion cannot meet the baby's require, the baby was mainly fed by other dairy products.

• Prolonged lochia rubra means lochia rubra lasted more than 1 week.

## Discussion

Although the traditional postpartum practices were still popular among women in China and Southeast Asia [17,18]. We know of no other studies in these areas that has attempted such work and reported similar results. This is the first such intervention study on postpartum dietary and health behaviors.

Our results show that the health and nutrition education intervention was associated with positive changes of postpartum practices. The intervention was successful in improving women's nutrition and health knowledge and some postpartum practices. An increased fruit intake was found in both rural and urban intervention groups. Significantly more women in the intervention groups give up the traditional behavior taboos (no bathing, no hair washing or teeth brushing) than those in the control groups. We have confirmed that traditional postpartum practices in both urban and rural community can be changed quickly by messages given by health professionals. Some of these knowledge, practice and health problem differences were small, but all were in the desired direction. We have shown that a nutrition and health education contact can have effect on postpartum practices even among lower educated, rural women. Cultural postpartum practices do not seem to be deeply ingrained.

The goal of this intervention was to encourage balanced diet and reduce unhealthy hygiene taboos. We stressed both individual behaviour change and information dissemination. And we had a cross-sectional survey of postpartum practices from the same province on which to base all our activities and messages [14]. We also emphasized to the health professionals about the existence of urban-rural difference, so that they could communicate with the participants effectively.

In the intervention process, we had emphasized on the nutrition advantages of milk and milk products. But the dairy consumption was still fairly low and leads to inadequate calcium intake. A possible explanation might be the overall consumption of milk and milk products are pretty low in Hubei, a great majority of people didn't used to drink milk every day, especially adults. Therefore, milk promotional strategies should be put forward to target specifically to women at child-bearing age.

Positive aspects of the present intervention include the nutrition knowledge of the women improved greatly, thus the intervention should optimally have positive effect on both the women themselves and their babies. The positive influences will help the mothers form a basis for good nutrition to be followed in later years.

The results of the study found that increased nutrition and health care knowledge did not lead to parallel dietary and health behavior changes. Several factors may explain this apparent incongruence. Nutrition and health care knowledge and attitudes may not have led to actual changes in behaviors. In china, the tradition to support a newly delivered woman and her baby for the first month after childbirth at home is still common. Most of the women had an elder female of the family such as her mother or mother-in-law as the support person. The elder female who takes care of the women may have hindrance the changes due to traditional believes.

The main problematic aspect of the study was the education intervention subjects aimed directly to the study women, yet "sitting month" was usually recognized as an important event in the family and the postpartum woman has been taken care by her mother or mother in-law. We should enlarge our intervention subjects not only to the pregnant women, but also to their family members [19]. The present results are promising enough to support continued investment in this intervention and attempts to improve it.

## Conclusion

Because of the successful outcomes, we conclude that this intervention has potential for further adaptation and development to other areas in China. We suggest that other channels, perhaps television, magazines and internet could be added to the intervention. If well-designed, these medias could potentially add a lively, modern, colorful and attractive channel for the messages to reach the families. However, the face-to-face contact of health professionals and the health education guidebook are still recommended as additional effective communication channels, as these were welcomed by most of the women.

## Competing interests

The authors declare that they have no competing interests.

## Authors' contributions

NL participated in data collection and analysis and drafted the manuscript. LM had made substantial contributions on the design of the study, participated in data collection, performed the statistical analysis and helped to draft the manuscript. XS supervised the design and execution of the study. LL and PY participated in coordination of the study and revised the manuscript. BC carried out the data collection and analysis. All authors read and approved the final manuscript.

## Pre-publication history

The pre-publication history for this paper can be accessed here:



## Supplementary Material

Additional file 1 **Survey on Dietary Behavior and Health Status during Women's Pregnancy and Postpartum Period.** The data provided is the questionnaire mentioned in the text. Click here for file
