# Supplementary material for: The effect of health and nutrition education intervention on women's postpartum beliefs and practices: a randomized controlled trial
Source: BMC Public Health. 2009 Feb 1;9:45. doi: 10.1186/1471-2458-9-45 (PMC2640472; doi:10.1186/1471-2458-9-45)
Supplement: Additional file 1 — Survey on Dietary Behavior and Health Status during Women's Pregnancy and Postpartum Period. The data provided is the questionnaire mentioned in the text. [file 1471-2458-9-45-S1.doc]

**Questionnaire on Nutrition and Health care Knowledge**

City: District: Street: Number:

Name: National Minority: Address:

Tel:

1. Which is the best source of calcium

①bone soup ②milk ③egg ④meat ⑤green vegetables ⑥don’t know

1. chicken soup is more nutritious than chicken

①yes ②no ③don’t know

1. Women can eat vegetables and fruit during puerperium

①yes ②no ③don’t know

1. Which food contains abundant vitamin C and carotene
2. rice ② green vegetables ③meat ④egg ⑤milk ⑥don’t know
3. Food sources of high quality protein

①meat ② rice ③soybean ④egg ⑤milk ⑥don’t know

1. Which food is iron-rich

①soybean ② green vegetables ③meat, animal liver ④egg ⑤milk ⑥don’t know

1. Kelp contains affluent iodine

①yes ②no ③don’t know

1. Colostrums should feed the infant

①yes ②no ③don’t know

1. Which is the best food for infants

①Formula milk ②breast milk ③milk ④don’t know

1. Bedroom should be ventilated everyday

①yes ②no ③don’t know

1. Relevant activity is better for recovery

①yes ②no ③don’t know

1. Sexual activity should not be initiated until 6 weeks after giving birth

①yes ②no ③don’t know

1. Vitamin D can be gained by basking in the sunshine

①yes ②no ③don’t know

1. Breastfeeding will not induce obesity of mother

①yes ②no ③don’t know

1. Women can brush teeth and take shower during puerperium

①yes ②no ③don’t know

1. What do you think of the importance of nutrition and health care knowledge?

①very important ②a little bit important ③not important ④don’t care

1. Where do you get these knowledge?

①from parents ②from relatives and friends ③from colleagues ④from books and magazines ⑤from television and radio ⑥from school ⑦the health education materials taken from hospitals ⑧directly from medical workers ⑨others______________

18. Which aspect is the most you interested?

①nutrition during pregnancy ②nutrition during puerperium ③infant feeding ④birth control ⑤disease prevention ⑥others______
